# Genetics of obesity: can an old dog teach us new tricks?

**DOI:** 10.1007/s00125-016-4187-x

**Published:** 2016-12-24

**Authors:** Giles S. H. Yeo

**Affiliations:** Medical Research Council (MRC) Metabolic Diseases Unit, University of Cambridge Metabolic Research Laboratories, Wellcome Trust-MRC Institute of Metabolic Science, Box 289, Addenbrooke’s Hospital, Cambridge, CB2 0QQ UK

**Keywords:** Appetite, Body weight, Food intake, Genetics, Hypothalamus, Melanocortin, Obesity, Review

## Abstract

At one level, obesity is clearly a problem of simple physics, a result of eating too much and not expending enough energy. The more complex question, however, is why do some people eat more than others? Studies of human and mouse genetics over the past two decades have uncovered a number of pathways within the brain that play a key role in the control of food intake. A prime example is the leptin–melanocortin pathway, which we now know greatly contributes to mammalian appetitive behaviour. However, genetic disruption of this pathway remains rare and does not represent the major burden of the disease that is carried by those of us with ‘common obesity’. In recent years, genome-wide association studies have revealed more than 100 different candidate genes linked to BMI, with most (including many components of the melanocortin pathway) acting in the central nervous system and influencing food intake. So while severe disruption of the melanocortin pathway results in severe obesity, subtle variations in these genes influence where you might sit in the normal distribution of BMI. As we now enter this ‘post-genomics’ world, can this new information influence our treatment and management of obese patients?

**Electronic supplementary material:**

The online version of this article (doi:10.1007/s00125-016-4187-x) contains a slideset of the figures for download, which is available to authorised users.

## Introduction

Obesity is arguably the greatest public health threat of the 21^st^ century. Its prevalence has increased dramatically over the past three decades in almost all developed economies, and it is becoming a significant burden in many other emerging economies. It isn’t only obesity that is the issue, but also the accompanying host of comorbidities, such as type 2 diabetes, cardiovascular disease, hypertension and certain cancers, which serve to amplify and exacerbate the problem. While our changing lifestyle and environment has undoubtedly driven the increase in obesity, there remains a large variation in the response to this ‘obesogenic’ environment. There is a powerful genetic component that underlies this variation, as revealed by its correlation with BMI (weight in kg/height in m^2^) in twin and adoption studies. These studies demonstrate that the heritability of fat mass is between 30% and 70% [1, 2]. As a consequence, genetic approaches can be leveraged to help characterise the underpinning physiological and molecular mechanisms controlling food intake and body weight, allowing us to understand how these may differ between lean and obese individuals.

Over the last 20 years, studies of human and mouse genetics have uncovered a number of pathways within the brain that play a key role in the control of food intake (reviewed in [3]). Of these, the best characterised mechanism is the hypothalamic leptin–melanocortin signalling pathway, which we now know greatly contributes to mammalian appetitive behaviour [4]. The vast majority of monogenic disorders that result in severe obesity in both mouse and man involve genetic disruption of this pathway [3].

**Fig. 1:**
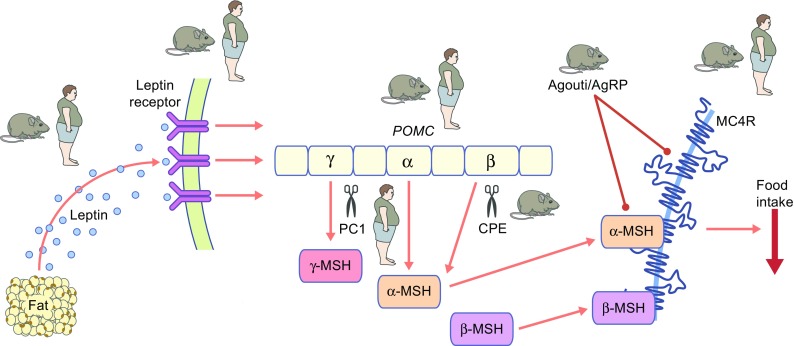
The old dog. POMC undergoes extensive post-translational processing to generate a range of smaller biologically active melanocortin peptides. Melanocortin signalling through MCR4 plays a key role in energy homeostasis. Disruption of the leptin–melanocortin pathway results in severe obesity in humans and mice. Existence of a human monogenic obesity syndrome or an obese mouse model is illustrated with an ‘obese man’ or an ‘obese mouse’. Lines ending in a circle indicate antagonism at a receptor. AgRP, agouti related peptide; CPE, carboxypeptidase E; PC1, prohormone convertase 1

There are two ‘old dogs’ that drive the narrative of this review; the first one is allegorical and represents the melanocortin pathway (Fig. 1), while the second refers to a study on the genetics of obesity in actual dogs. Here, I will briefly chart the history of the melanocortin pathway in energy homeostasis and then highlight some contemporary studies that reveal ‘new tricks’ emanating from the ‘old dog’. I will then discuss how these rare genetic disorders that result in severe obesity have been able to inform more common forms of obesity, and explore whether this new information can influence our treatment and management of obese patients.

## Old dog: the leptin–melanocortin pathway

The discovery of leptin in 1994 [5] heralded the modern era of genetic and mechanistic obesity studies, providing the first direct evidence for a feedback loop, of sorts, that could control food intake. In 1997 came the first report that two young cousins with severe early onset obesity harboured mutations in the gene encoding leptin (*LEP*), confirming that this system was relevant across mammalian species [6]. Leptin is secreted by white adipose tissue and is a key indicator of fat mass. This led many to assume that leptin was a ‘satiety factor’, existing to let the brain know when there was too much fat and, thus, when to stop eating. In fact, the moniker ‘leptin’ comes from the Greek term ‘leptos’, meaning thin. In 1999 came what appeared to be the proof of this hypothesis: the reported dramatic reversal of obesity in congenital leptin-deficient children that were treated daily with recombinant leptin [7]. In the intervening years, however, it has become clear that while patients with leptin deficiency are exceptionally sensitive to leptin administration, anyone with an intact system will not respond to leptin, certainly with regards to food intake and loss of bodyweight [8]. We now know that leptin is not a satiety signal, but rather a signal of starvation [9]. For the vast majority of us, leptin acts in the background, letting our brain know how much fat we are carrying, which is a critical piece of information because how much fat we have is, put simply, reflective of how long we would survive without food. Adding more exogenous leptin changes little because we still have a surfeit of fat. However, in times of starvation when fat levels plummet, leptin levels drop as well, turning on the starvation response [9].

The melanocortin pathway is one of the key effector mechanisms of leptin signalling in the brain (Fig. 1). The central and eponymous component to the pathway is pro-opiomelanocortin (POMC), which undergoes extensive post-translational processing to generate a range of smaller biologically active melanocortin peptides, as well as β-endorphin and β-lipotrophin. The melanocortin peptides are agonists for five melanocortin receptors, melanocortin 1 receptor (MC1R) to –MC5R, through which they mediate many different functions (reviewed in [3, 4]); melanocortin peptide signalling through the MC4R plays a key role in energy homeostasis. Indeed, both *Pomc*/*POMC*-deficient mice [10] and humans [11] develop hyperphagia and obesity, and genetic deletion of *Mc4r*/*MC4R* in mice [12] and humans [13, 14] results in severe hyperphagic obesity. In fact, to this day *MC4R* mutations remain the most common monogenic form of obesity, with pathogenic mutations found in up to 5% of cases of severe childhood obesity [15] and up to 1% of the general population with a BMI >30 kg/m^2^ [16]. In particular, the degree of receptor dysfunction (as measured by *in vitro* assays) can be used to predict the amount of food eaten by the patient harbouring that particular mutation during a test meal [15].

## New tricks for POMC

**Fig. 2:**
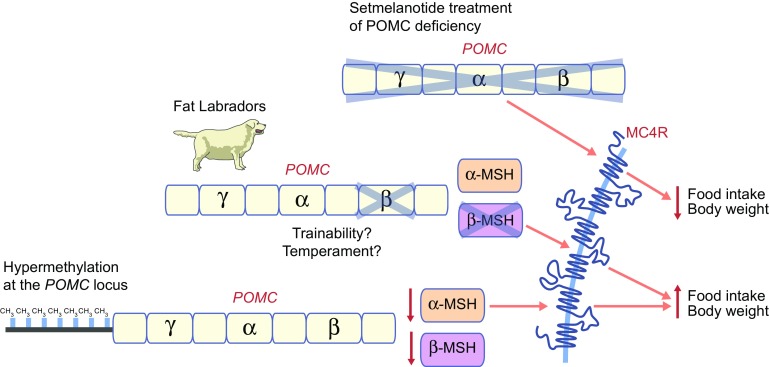
New tricks. Recent findings in POMC biology have demonstrated that: (1) partial deletion of *POMC* results in obesity in Labrador Retrievers because of increased food motivation [19]; (2) hypermethylation at the *POMC* locus and thus lowering of melanocortinergic tone, is associated with obesity [22, 23]; and (3) human POMC deficiency may be treated with setmelanotide, an MC4R agonist, resulting in reduced food intake [24]

In recent years, new findings have emerged to provide an increasingly more nuanced view of the melanocortin pathway and of POMC in particular. Some of these findings are outlined in Fig. 2 and discussed in more detail in this section.

### Fat Labradors (Flabradors)

The investigation of humans with *POMC* mutations that result in specific dysregulation of β-melanocyte-stimulating hormone (MSH) [17, 18] suggests that β-MSH is important for controlling food intake and body weight in man. However, although many of the mechanistic details regarding melanocortin signalling were elucidated using rodent models, rodents lack the proximal dibasic cleavage site necessary for the production of β-MSH. Thus, the in vivo study of the role of β-MSH in appetitive behaviour has been, until recently, quite limited. As an alternative to rodent models, dogs that carry a 14 bp deletion in *POMC*, which disrupts only the β-MSH and β-endorphin coding sequences [19], represent a ‘clean’ model of β-MSH/β-endorphin deficiency and thus provide an ideal opportunity to study the role of these hormones in feeding behaviour.

All dogs descend from the grey wolf and were domesticated some 12,000–20,000 years ago. Modern dog breeds, however, originated only relatively recently from a small number of founder animals. Because of this ‘bottlenecking’ phenomenon, dissecting the genetic basis of canine traits is more amenable than in other more outbred species, such as humans [20]. We have been interested in studying the genetics of obesity in dogs and began with Labrador Retrievers, which are more food-motivated than other breeds [21]. We found that 20% of Labradors were heterozygous and 2% were homozygous for the 14 bp deletion in *POMC* and that this mutation is associated with greater food motivation and increased body weight [19]. In addition to Labradors, 38 other dog breeds were also screened for this mutation, but it was only found in the closely related Flat-Coated Retriever, in which it was also associated with increased adiposity and food motivation [19] .

What is particularly intriguing is that while 22% of pet Labradors carried the *POMC* deletion in our study, 80% of Labradors that become guide dogs also have the deletion. Because guide dogs are essentially responsible for a human life, they are very highly selected for temperament and trainability, and ‘positive reinforcement’ with food rewards plays a key role during training. We found the allelic frequency at the *POMC* locus to be significantly out of Hardy–Weinberg equilibrium in guide dog breeding stocks, supporting our hypothesis that dogs carrying the *POMC* deletion may be more likely to be selected [19]. Thus, although we began the study to understand the genetics of obesity and food motivation, we have also begun to uncover tantalising clues about other more complex traits, those of temperament and trainability.

### POMC epigenetics

Another ‘new trick’ has emerged from two reports from Krude’s group, providing evidence that the risk for common diseases may also depend on variation in epigenetic marks at specific loci of the *POMC* gene [22, 23]. In an initial study in 2012, Kühnen and colleagues identified two CpG islands at the *POMC* locus and determined their methylation status in lean and obese children. They found that there was a significant increase in the methylation of CpGs at the intron 2/exon 3 boundary of the *POMC* gene in obese children vs lean children [22]. This hypermethylation at exon 3 interferes with the binding of the p300 transcriptional enhancer, resulting in reduced expression of *POMC*. This was the first DNA methylation variant to be associated with the risk for obesity. In a second study, the same group showed that methylation in a variably methylated region (VMR) in *POMC*, as opposed to individual CpGs, was strongly associated with individual BMI. They found that multiple factors could trigger *POMC* VMR methylation in the early embryo and that BMI is directly related to the level of methylation as a continuous trait [23].

While these findings have emerged from the same group and thus have yet to be replicated, conceptually, increased methylation states in both cases most likely represents a more subtle lowering of melanocortinergic tone, therefore linking it to an appropriately subtle increase in BMI. These findings are consistent with the previously mentioned finding that the magnitude of MC4R dysfunction can predict food intake at an ad libitum test meal [15], emphasising that the melanocortin system does not act in a binary on/off fashion but as a rheostat.

### Treatment of POMC deficiency

Aside for treatments for leptin deficiency, there have been no effective pharmacological agents available for the hyperphagia and obesity that characterises the deficiency in melanocortin signalling; that is, until this year. In an investigator-initiated, open-label study, Kühnen and colleagues, in collaboration with Rhythm Pharmaceuticals, treated two POMC-deficient patients with setmelanotide, a new MC4R agonist [24]. The patients demonstrated a sustained reduction in hyperphagia and dramatic weight loss (Patient 1: 51.0 kg after 42 weeks; Patient 2: 20.5 kg after 12 weeks). This degree of pharmaceutically induced weight loss has not been seen since leptin-deficient children were first treated with leptin [7] and, hence, these findings have energised the field. Given the role of MC4R signalling in the modulation of blood pressure [25], the main issue with use of previous MC4R agonists has always been the accompanying increase in blood pressure, which is clearly not ideal in obese patients. Setmelanotide, however, appears to achieve its effects on food intake and weight loss without the pressor effects [24] for reasons that are yet to be determined. However, since the dramatic reduction of food intake by patients indicates a powerful central effect, one could perhaps speculate that this compound only reaches the medial basal hypothalamus, where appetite is primarily regulated [3], and does not affect areas of the brain where blood pressure is regulated. Despite the beneficial outcomes with setmelanotide use, however, there was a substantial darkening of skin pigmentation and hair colour in the two patients that indicated measurable activation of the peripheral MC1Rs [24] and a potential for increased risk of skin cancer. Nonetheless, the benefits undoubtedly outweigh the risks for the two POMC-deficient patients, who are still both being scrutinised very closely indeed, in this ongoing study.

The successful use of setmelanotide has opened doors for clinical trials of treatments for other monogenic deficiencies of the leptin–melanocortin pathway, such as *MC4R*-deficiency (which occurs in a far larger cohort than *POMC* deficiency) and *leptin receptor* deficiency the sufferers of which, for obvious reasons, are resistant to leptin treatment. These studies are currently ongoing, and we in the field are eagerly awaiting the results. The eventual goal is clearly to trial setmelanotide for the treatment of more common forms of obesity. Before this happens, however, its mechanisms of action, both central and peripheral, will need to be defined in more detail.

## Complex simplicity

What about common polygenic obesity then? It has been nearly 10 years since the first obesity genome-wide association studies (GWAS) were published [26], with many more being published in the intervening period (reviewed in [27]). Was it worth the investment? Are we getting any closer to revealing the underlying biology of obesity?

**Fig. 3:**
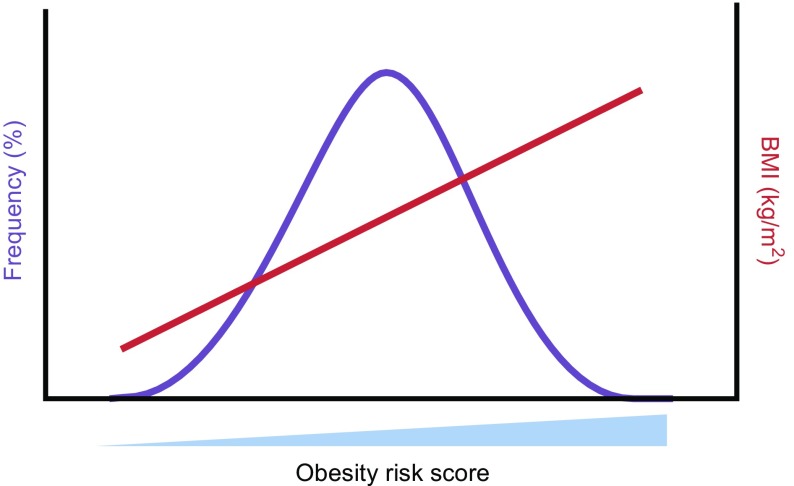
Obesity risk score is directly related to BMI in the population. Schematic figure illustrating that an obesity risk score calculated using GWAS-identified SNPs is directly related to BMI (in red). The frequency (in purple) of obesity risk score increases from left to right, following the normal distribution in the population

After the initial hype and over-expectation, which was followed by the inevitable negativity and swing in the opposite direction, the dust has now settled sufficiently to allow a cold hard look at the information on common obesity that has been provided by GWAS. While knowledge of actual mechanisms is still thin on the ground, a surprisingly cogent narrative has emerged regarding the genetic architecture of common obesity: (1) the genes that lie closest to the single nucleotide polymorphisms (SNPs) associated with waist:hip ratio (i.e. where your fat is deposited), tend to be primarily expressed in fat [28]; (2) the genes that lie closest to the SNPs associated with BMI (i.e. how much fat you have), are primarily expressed in the central nervous system [29]. In fact, SNPs at more than 100 robust and well-replicated loci have now been found to influence BMI. From these SNPs, one can generate an obesity risk score, with each SNP having a possible score of 2 (homozygous for the risk allele), 1 (heterozygous) or 0 (homozygous for the non-risk allele). When plotted against a large enough population, the risk score follows a normal distribution and an increasing risk score is directly related to increasing BMI [29] (Fig. 3).

What of our central theme, our ‘old dog’? Well, a closer look at the identities of some of these genes reveals many members of the leptin–melanocortin pathway; amongst them *POMC* and *MC4R* [29]. So what is clear is that while the brain leptin–melanocortin pathway is central to mammalian food intake control with genetic disruption resulting in extreme obesity, subtle variations in these same genes influence an individual’s position in the normal distribution of BMI.

## The $64 million question

As we now enter this ‘post-genomics’ world, can this new (and not so new) genetic information that has been discussed here influence the treatment and management of obese patients? Are we any closer to ‘precision medicine’ for obesity? In short, the answer at the moment is ‘it depends’. For monogenic forms of obesity, the answer is a resounding ‘yes’. We can now leverage genomic medicine to identify and potentially treat the 1% of the population with a BMI >30 kg/m^2^ that harbour genetic mutations within the leptin–melanocortin pathway. The use of precision medicine to inform treatment of common polygenic obesity, however, is another story entirely, as the predictive value of these SNPs and risk scores are still very poor for any given individual. This is likely because of a combination of the small effect size, together with the thus far unquantified broader genetic and environmental influences.

For one thing, it is amazing that in a field that uses such cutting edge technology, our ability to accurately and precisely measure food intake and energy expenditure on a large scale (in tens to hundreds of thousands of people) is still very primitive. We will have to rectify this problem if we truly want to bring the field of nutrigenomics from the Stone Age to the 21^st^ century. That being said, this has not stopped commercial entities from stepping in and offering ‘nutrigenomics’, ‘exercise genomics’, ‘personalised weight loss plans’ and all manner of other ‘omics’-based predictions. Clearly genetic technology has reached the stage where it is no longer the limiting factor and pretty much anybody with a genetic analyser can accurately produce genetic information. The problem of course is in the interpretation and these charlatans (that’s what they are) are fundamentally misunderstanding the difference between risk and prediction. However, the genie is now out of the bottle and any of us can now pop down to our local drugstore or click online, pay 100–200 £/$/€, and obtain our genetic profile. This should be motivation enough for the scientific community to get their act together and provide some real genetic interpretation for the public.

## Summary

In conclusion, the fact that the leptin–melanocortin pathway is a key controller of mammalian appetitive behaviour is now an ‘old dog’ in this field. Yet, new research tricks continue to allow us to obtain a deeper and more nuanced understanding in this area. The current focus of pharmacological therapies is aimed at individuals with deficiencies in the leptin–melanocortin pathway (see text box: summary). However, genetic disruption of this pathway still remains rare, whereas the majority of individuals suffer from ‘common obesity’, which represents the major public health threat. Therefore, while we have made enormous strides in our understanding of the mechanisms controlling food intake and body weight, further advances must be urgently made to enable the optimal management and treatment of common obesity.
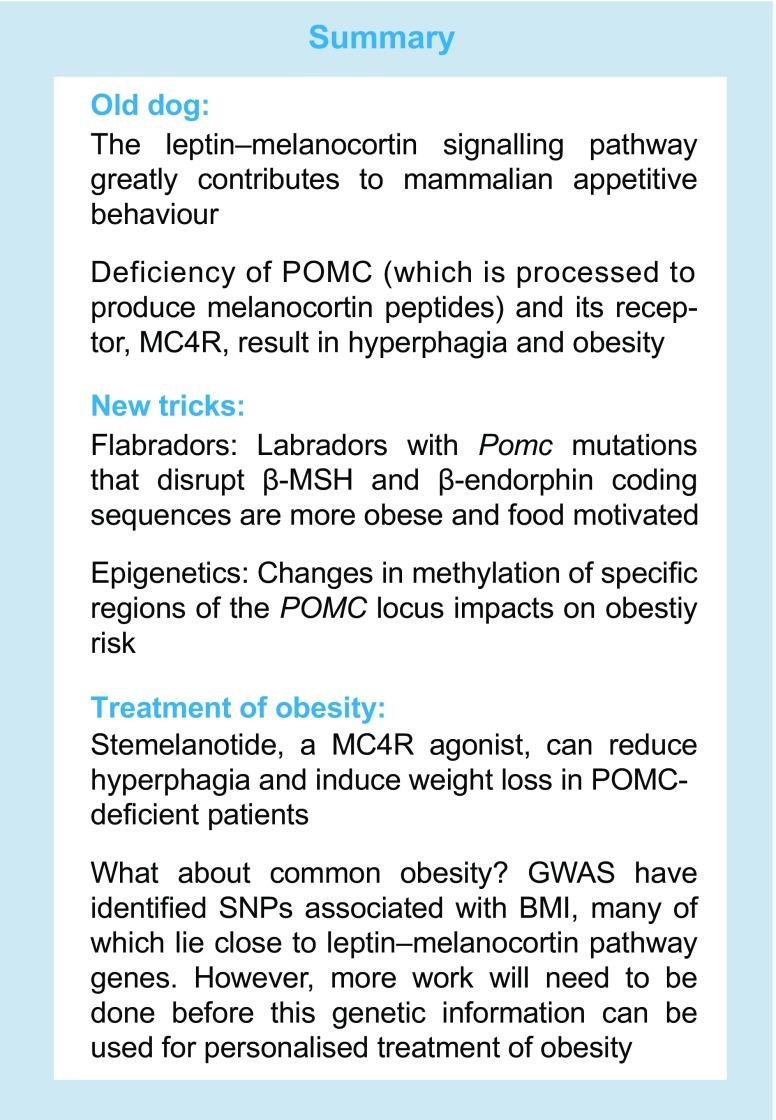


## Electronic supplementary material

Below is the link to the electronic supplementary material.

ESM 1 (PDF 289 kb)

## Abbreviations

GWAS: Genome-wide association studies
MC*n*R: Melanocortin *n* receptor
MSH: Melanocyte-stimulating hormone
POMC: Pro-opiomelanocortin
SNP: Single nucleotide polymorphism
VMR: Variably methylated region
