# Supplementary figures and images for: Genetics of obesity: can an old dog teach us new tricks?
Source: Diabetologia. 2016 Dec 24;60(5):778–83. doi: 10.1007/s00125-016-4187-x (PMC6518377; doi:10.1007/s00125-016-4187-x)

# The old dog

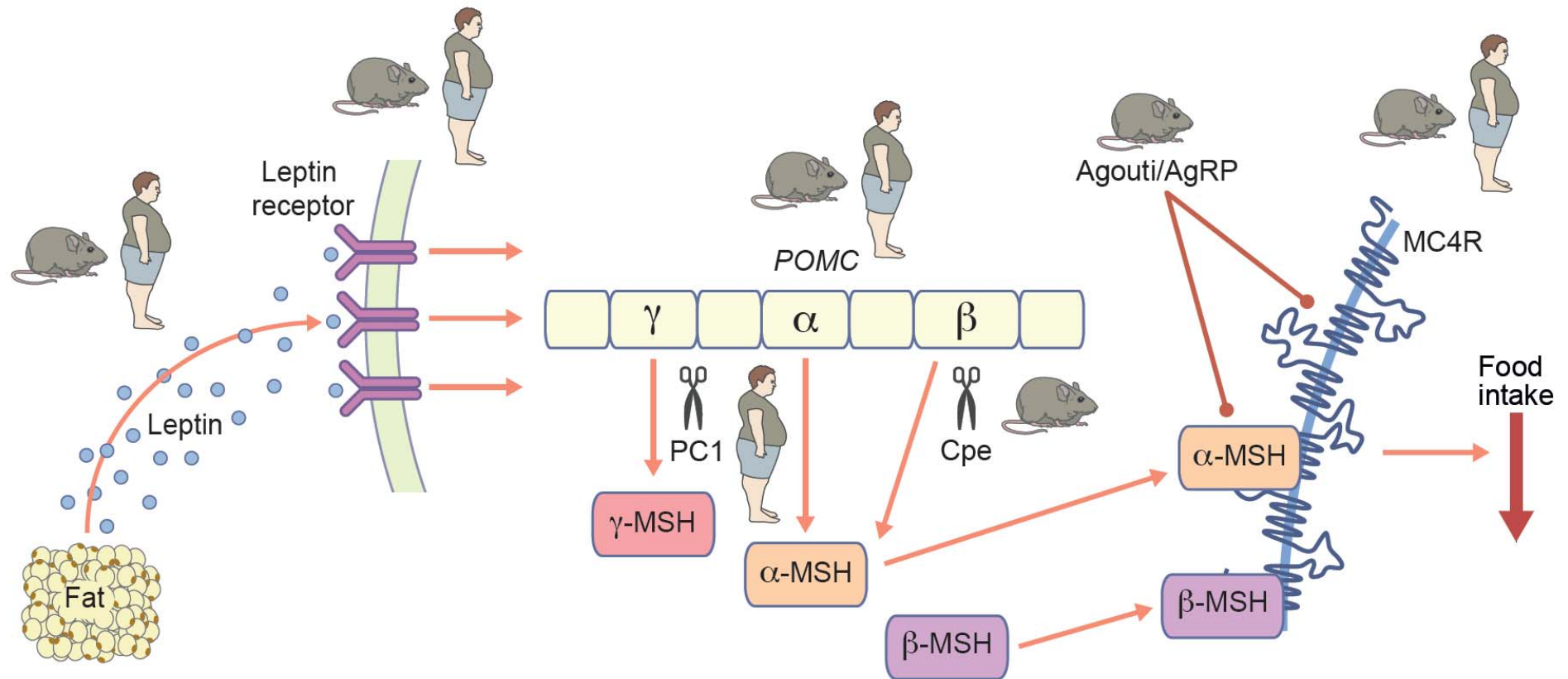

# New tricks

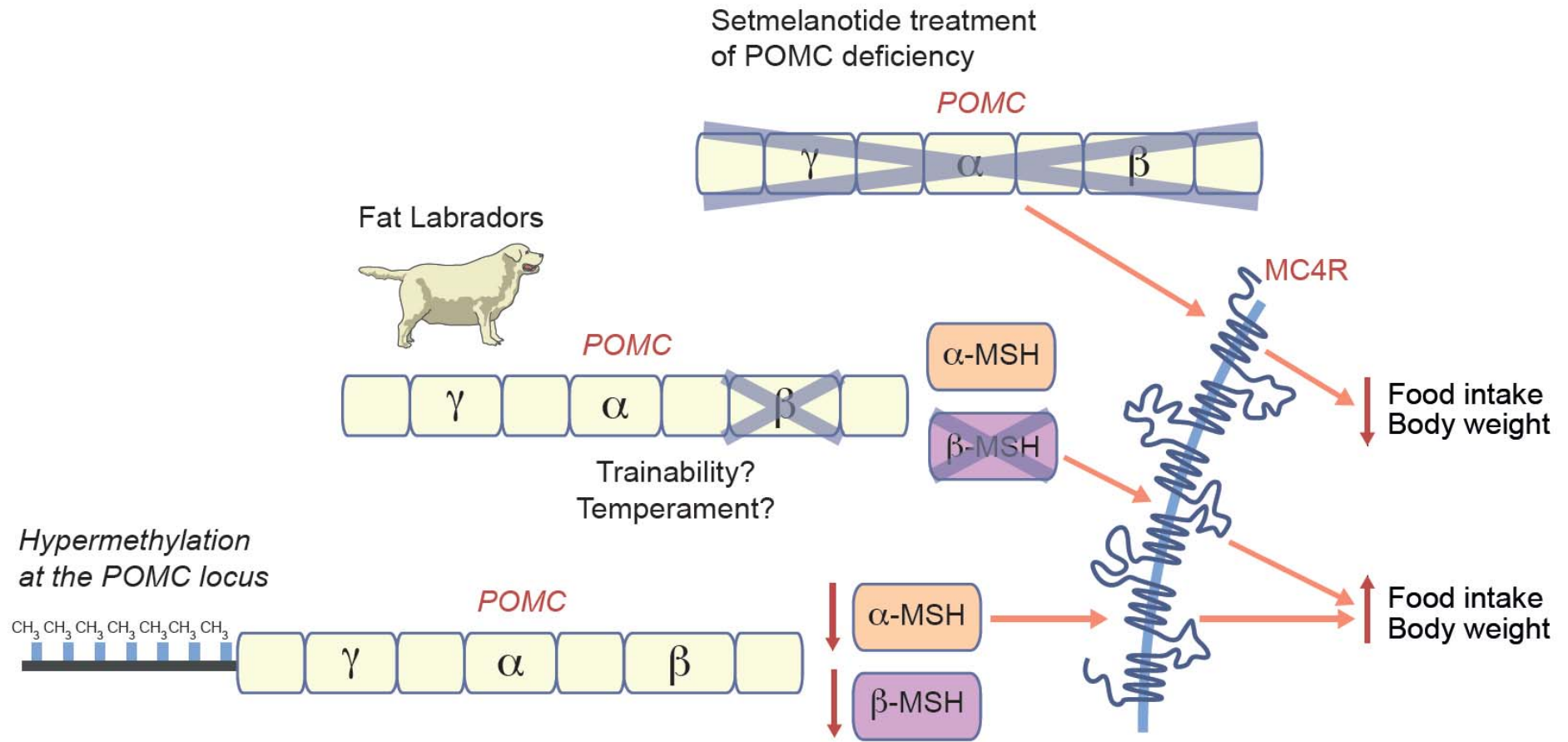

# Obesity risk score is directly related to BMI in the population

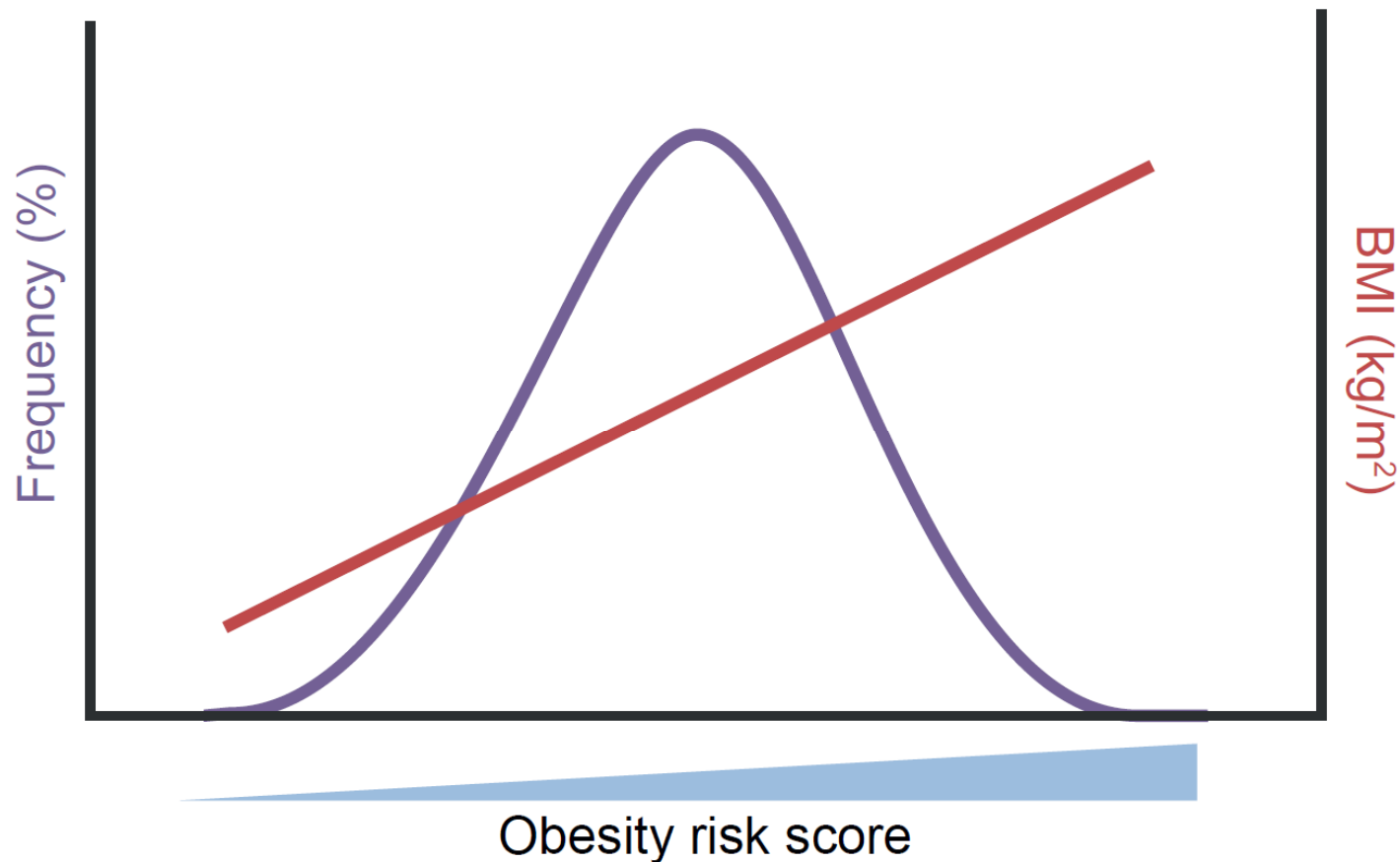

Supplement: Supplementary file 1 — (PDF 289 kb) [file 125_2016_4187_MOESM1_ESM.pdf]
